# Pyramidal-Shaped Costal Cartilage Columellar Strut Graft with Half-Harvest Technique for Augmentation Rhinoplasty: A Novel Approach to Tip Mobility Preservation

**DOI:** 10.3390/jcm15134985

**Published:** 2026-06-26

**Authors:** Hyo Heon Kim, Hee Jun Son

**Affiliations:** Department of Plastic and Reconstructive Surgery, Gachon University Gil Medical Center, Incheon 21565, Republic of Korea; jjun@gilhospital.com

**Keywords:** augmentation rhinoplasty, costal cartilage, columellar strut graft, pyramidal graft, floating tip, half-harvest technique, nasal tip mobility, rib cartilage rhinoplasty

## Abstract

**Background:** Costal cartilage is the preferred structural material for augmentation rhinoplasty when robust and durable tip support is required. However, conventional full-thickness harvest is associated with significant donor-site morbidity, and commonly employed rigid fixation strategies—such as the septal extension graft—substantially restrict postoperative nasal tip compliance. The present study introduces a novel two-component technique combining a half-harvest costal cartilage procurement method with a pyramidal-shaped columellar strut graft anchored on the floating-tip principle, with the objective of maintaining postoperative nasal tip flexibility while providing structural support following augmentation rhinoplasty. **Methods:** A retrospective review was performed of consecutive patients who underwent primary or revision augmentation rhinoplasty using the pyramidal costal cartilage columellar strut graft technique by a single surgeon between June 2018 and February 2026. The medial half of the conjoined costal cartilage at the seventh, eighth, or ninth rib was procured via a half-harvest approach, preserving the lateral cortex and perichondrium to minimize donor-site morbidity and potential cartilage regeneration was considered a theoretical benefit. The harvested cartilage was carved into a pyramidal columellar strut and secured to the anterior nasal spine using a floating fixation construct; the inferior base of the strut was rigidly fixed to the nasal septum and anterior nasal spine with a minimum of three PDS 5-0 sutures, while the superior portion remained free to preserve physiologic nasal tip mobility. Adjunctive cap and shield grafts, perichondrial wrapping, and dermal fat grafts were employed as indicated. Primary outcomes included nasal tip projection, postoperative tip mobility, donor-site morbidity, and surgical complication rates. **Results:** Favorable clinical observations of maintained tip projection were noted throughout follow-up. Manual postoperative examination suggested preservation of tip flexibility in most patients; however, no validated objective mobility assessment tool was available. The revision rate for clinically significant tip deviation was low. No major donor-site adverse events—including pneumothorax or rib fracture—were encountered. Postoperative chest wall pain was minimal and transient, with most patients resuming daily activities within one week of surgery. **Conclusions:** The pyramidal-shaped costal cartilage columellar strut graft with half-harvest technique is a novel, biomechanically informed, and technically reproducible approach to augmentation rhinoplasty that was developed to address donor-site morbidity and postoperative tip rigidity, two commonly recognized limitations of conventional costal cartilage rhinoplasty: donor-site morbidity and postoperative nasal tip rigidity. Preservation of the lateral cortex and perichondrium during procurement may contribute to reduced postoperative donor-site discomfort, accelerates functional recovery, and may promote endogenous cartilage regeneration over time. The anatomically derived pyramidal strut geometry, combined with floating fixation to the anterior nasal spine, was designed to approximate the native columellar architecture, enabling consistent preservation of physiologic nasal tip mobility. The present series demonstrated a favorable safety profile with a low overall complication rate and an absence of major donor-site adverse events. Prospective studies with validated objective outcome measures are required to confirm these findings, to delineate the optimal patient selection criteria, and to establish evidence-based long-term outcome benchmarks for this technique.

## 1. Introduction

Costal cartilage augmentation rhinoplasty is widely recognized as a reliable and durable approach to structural nasal reconstruction, particularly in patients with insufficient autologous septal or auricular cartilage reserves and in those presenting for complex revision surgery [1,2]. The rib cartilage offers the volume, structural integrity, and carving versatility required for comprehensive nasal framework reconstruction [3,4], encompassing dorsal augmentation, nasal tip projection, and columellar support [5].

Conventional approaches to costal cartilage tip support—including septal extension grafts and the tongue-in-groove technique—reliably establish tip projection and rotation [6,7]; however, these strategies achieve structural rigidity at the cost of natural nasal tip compliance [8]. Firm suture fixation of the medial crura to the caudal septum abolishes the intrinsic spring-like mobility of the nasal tip—a quality that is a fundamental determinant of both natural aesthetic appearance and normal nasal function [9]. Postoperative tip stiffness represents one of the most frequently reported sources of patient dissatisfaction following costal cartilage rhinoplasty, yet its prevention remains an insufficiently addressed objective in the surgical literature [10].

Donor-site morbidity represents a second major limitation of conventional costal cartilage rhinoplasty [11]. Standard full-thickness rib harvest necessitates complete transection of the cartilaginous rib segment [12] and is associated with postoperative chest wall pain, impaired trunk mobility, and a non-negligible risk of pneumothorax [13,14]. These considerations may adversely affect patient acceptance of the procedure and substantially prolong the postoperative recovery course.

The floating-tip columellar strut concept—in which the caudal end of the strut is stabilized at the anterior nasal spine without rigid attachment to the caudal septum—was introduced as a strategy to preserve a greater degree of postoperative tip flexibility while providing a reliable degree of columellar support and tip projection [15].

The purpose of this study is to delineate the operative principles, technical execution, and clinical outcomes of this technique in a consecutive single-surgeon patient series.

## 2. Materials and Methods

### 2.1. Study Design and Patients

This retrospective cohort study was conducted in accordance with the ethical principles of the Declaration of Helsinki and received formal ethical approval from the Institutional Review Board of Gachon University Gil Medical Center. The study population, however, encompasses all patients who underwent the described procedure under the care of the author (H.H.K.) between 2018 and 2026 using this standardized surgical technique, and is not limited to patients treated at Gachon University Gil Medical Center alone; it includes patients managed at prior institutional affiliations and in private practice settings throughout this period. Written informed consent was obtained from all patients prior to surgery. The study cohort comprised a consecutive series of patients who underwent primary or revision augmentation rhinoplasty using the pyramidal costal cartilage columellar strut graft technique performed exclusively by the senior author. Patients with a postoperative follow-up duration of less than 6 months were excluded from the analysis.

### 2.2. Donor-Site Selection and Incision Planning

Costal cartilage was procured from the most proximal cartilaginous segment of the conjoined seventh, eighth, or ninth rib complex. A skin incision of approximately 2 cm was placed within medial side of the inframammary fold; in female patients, this was positioned within medial side of the inferior mammary crease, where it is naturally concealed beneath the breast mound and fully obscured by a standard brassiere. In the author’s experience, this incision consistently evolves into a hypopigmented, discontinuous, and clinically imperceptible scar by approximately 6 months postoperatively.

### 2.3. Half-Harvest Technique

**Figure 1:**
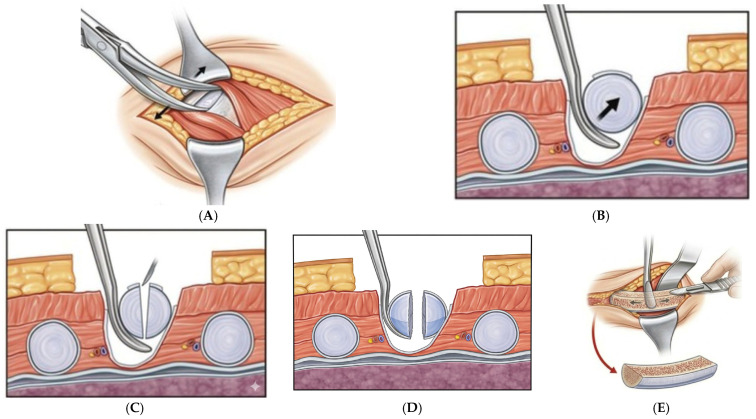
Schematic illustration of the half-harvest costal cartilage procurement technique. (**A**) A 2-cm skin incision is placed at the medial aspect of the inframammary fold, followed by subperichondrial dissection. (**B**) Exposure of the conjoined costal cartilage is performed while preserving the lateral cortex and lateral perichondrium. (**C**) Longitudinal splitting of the cartilage is carried out at its midpoint with protection of the underlying structures. (**D**) The medial half of the cartilage is separated from the preserved lateral segment. (**E**) Harvest of the medial half-segment is completed, leaving the lateral cortex and perichondrium intact. This approach was designed to obtain sufficient graft material while preserving structural continuity of the donor site. Declare; the authors used Gemini (free version, Google, https://gemini.google.com/app) for the purposes of generating the image for this Figure.

A 2-cm skin incision was placed at the medial aspect of the inframammary fold, and subperichondrial dissection was performed to expose the conjoined costal cartilage while preserving the lateral cortex and lateral perichondrial layer (Figure 1A,B). The cartilage was then divided longitudinally at its midpoint under direct vision, with careful protection of the underlying intercostal structures (Figure 1C). The medial half of the cartilage column was subsequently separated from the preserved lateral segment and harvested as the graft material, leaving the lateral cortex and perichondrium entirely intact at the donor site (Figure 1D,E). This approach was designed to obtain sufficient structural graft material while maintaining the continuity of the donor-site rib segment.

### 2.4. Graft Design: Pyramidal Configuration

**Figure 2:**
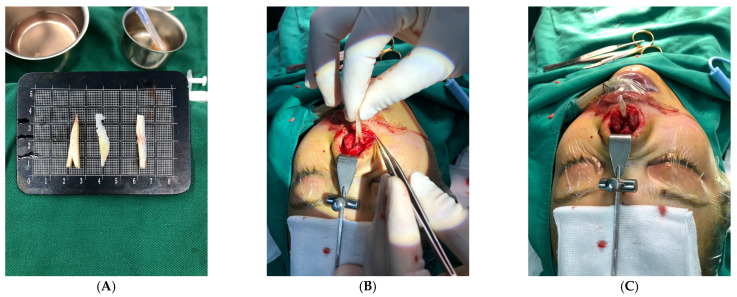
Intraoperative preparation and fixation of the pyramidal costal cartilage columellar strut. (**A**) Harvested costal cartilage grafts demonstrating the pyramidal columellar strut (**left**), cap and shield grafts (**center**), and derotation graft (**right**). The pyramidal strut was designed with a broad basal platform and progressive superior tapering to approximate the native contour of the columellar complex. (**B**) Preparation of the anterior nasal spine (ANS). Prior to fixation, the caudal portion of the ANS is reshaped into an L-shaped configuration measuring approximately 5 mm in both horizontal and vertical dimensions to facilitate stable seating of the graft base. (**C**) Final positioning of the pyramidal strut. The basal portion of the graft is secured to the prepared ANS using 5-0 PDS sutures, creating a fixed foundation while allowing the distal portion of the construct to function without rigid septal fixation. This configuration was designed to provide structural support while maintaining postoperative tip flexibility.

Upon completion of harvest, the costal cartilage was carved into a three-dimensional pyramidal strut. The geometric rationale underlying this design derives directly from the native anatomy of the columellar complex: in its physiologic state, the columella narrows progressively from its base at the anterior nasal spine toward the nasal tip, assuming its widest cross-sectional dimension inferiorly and its narrowest superiorly. Precise replication of this anatomical tapering within the graft construct is therefore fundamental to achieving a columellar profile that is both biomechanically appropriate and aesthetically coherent (Figure 2A).

The inferior base of the pyramidal strut—the surface that articulates directly with the anterior nasal spine—was sculpted to a width of approximately 10 mm. A precisely dimensioned triangular notch was carved into the base to conform to the bony profile of the anterior nasal spine, establishing an interlocking mortise-and-tenon engagement that confers inherent three-dimensional rotational stability at the strut-spine interface (Figure 2B). Before securing the pyramidal strut to the anterior nasal spine (ANS), we resected the caudal part of the ANS into an ‘L’ shape—measuring 5 mm horizontally and vertically—to ensure optimal seating of the strut. We then securely sutured the horizontal and vertical dimensions of the L-shape using 5-0 PDS sutures manufactured by Ethicon (Somerville, NJ, USA). This configuration creates a firmly fixed base acting as a rotational pivot, while maintaining the nasal tip in a freely floating state (Figure 2C).

The strut tapered progressively from the base toward the apex, where it was dimensioned to seat securely between the medial crura and transmit the desired tip projection vector.

In cases requiring augmented columellar length or corrective tip derotation, the intrinsic curvature of the resected costal cartilage segment was exploited as a derotation graft, harnessing the material’s natural spring-force properties to supplement the structural and directional support of the pyramidal strut.

### 2.5. Adjunctive Grafts and Soft Tissue Coverage

Cap grafts and shield grafts, carved from residual costal cartilage, were employed as indicated to refine nasal tip contour, augment supratip definition, and delineate the infratip lobule. Final coverage of the composite graft construct was achieved through a structured multilayer soft tissue technique. The perichondrium procured at the time of costal cartilage harvest served as the primary enveloping layer; this was supplemented, when indicated, by local soft tissue advancement or by an autologous fascial graft harvested from the temporal region. This stratified coverage approach functions to attenuate surface irregularities of the underlying graft, minimize the risk of visible contour deformity through the skin envelope, and facilitate early fibrovascular adherence between the graft and its surrounding soft tissue bed.

### 2.6. Postoperative Protocol

Postoperative nasal tip immobilization is a critically important element of the aftercare protocol specific to this technique. Because the floating-tip columellar strut achieves stability through fibrovascular ingrowth and progressive soft tissue adherence—rather than through rigid suture fixation to the septum and ANS—premature mechanical loading of the nasal tip before adequate graft-tissue integration has occurred poses a substantive risk of graft displacement or malrotation. Accordingly, all patients were instructed to refrain from forceful digital manipulation or lateral compression of the nasal tip for a minimum of 4 weeks postoperatively. A custom-fitted thermoplastic nasal dorsal splint was maintained for 2 weeks following surgery. Patients were additionally counseled regarding gentle wound hygiene and the importance of avoiding nasal compression during sleep throughout the early healing period.

### 2.7. Outcome Assessment

**Figure 3:**
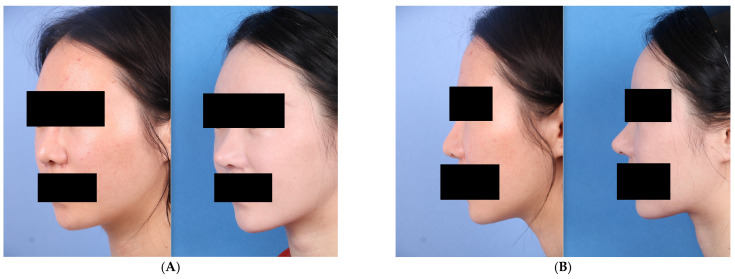
Representative clinical case at 12 months following augmentation rhinoplasty using the pyramidal costal cartilage columellar strut with the half-harvest technique. Left panels show the preoperative appearance and right panels show the 12-month postoperative results. (**A**) Oblique views demonstrating improvement in tip projection and columellar contour. (**B**) Lateral views demonstrating maintenance of tip projection and dorsal-tip relationship during the follow-up period. Clinically observable tip flexibility was noted on manual examination at 12 months postoperatively. These findings suggest satisfactory aesthetic and structural outcomes in this representative case.

Clinical outcomes were evaluated using standardized photographic documentation in the frontal, lateral, and basal projections, obtained initially and annually thereafter (Figure 3). Nasal tip projection was assessed using standardized photographic evaluation by the operating surgeon. Quantitative E-line measurements were not consistently available for the entire retrospective cohort. Postoperative outcomes were assessed at defined intervals: 1 week, 1 month, 3 months, 6 months, 1 year; nasal tip mobility was evaluated by the operating surgeon using a standardized manual examination protocol consisting of graded inferior and superior displacement of the nasal tip; findings were compared with preoperative baseline assessment. Donor-site outcomes—including incision scar maturation, residual chest wall pain on a visual analog scale, and any functional limitation of trunk mobility—were recorded at each follow-up visit. Complications, including nasal tip deviation, graft or implant exposure, surgical site infection, and donor-site adverse events, were prospectively documented. Revision surgery requirements were recorded and classified by indication.

## 3. Results

A total of 200 consecutive patients underwent augmentation rhinoplasty with the pyramidal costal cartilage columellar strut graft technique during the study period. Baseline demographic characteristics and operative parameters for the study cohort are summarized in Table 1.

Satisfactory nasal tip projection was achieved in the vast majority of patients and was maintained without significant change throughout the follow-up period. Standardized clinical assessment of postoperative tip mobility suggested maintenance of clinically appreciable tip flexibility on manual examination, supporting favorable clinical observations consistent with the intended floating-tip design of the floating-tip fixation strategy. Patients consistently described the nasal tip as exhibiting a natural tactile quality on palpation and during animated facial expression, in notable contrast to the rigid, immobile character commonly reported following septum-anchored fixation techniques. Intraoperative demonstration of unrestricted columellar mobility following pyramidal strut placement is provided in Video S1, Supplemental Digital Content (see Supplemental Digital Content S1, available online with this article, which demonstrates free multidirectional mobility of the pyramidal columellar strut at the anterior nasal spine prior to soft tissue closure). Long-term preservation of physiologic nasal tip mobility at 1 year postoperatively is demonstrated in Video S2, Supplemental Digital Content (see Supplemental Digital Content S2, available online with this article, which shows free nasal tip rotation and natural elastic recoil upon manual upturn displacement, confirming sustained tip compliance at 12 months following surgery).

Among the operative cohort, 5 patients (2.5%) presented with mild postoperative nasal tip deviation on follow-up examination. Of these, 4 cases (2%) were determined to fall within the clinically acceptable range of symmetry on blinded standardized photographic analysis and required no further intervention. The remaining 1 cases (0.5%) demonstrated clinically significant tip asymmetry and were managed with revision surgery.

Donor-site outcomes were uniformly favorable throughout the series. No cases of pneumothorax, intraoperative rib fracture, or wound dehiscence were recorded. The 2-cm incision achieved an inconspicuous, well-healed scar appearance by 6 months postoperatively in the majority of patients. Postoperative chest wall pain was characteristically mild and self-limiting, with resolution observed within the first postoperative week in most cases. No patient reported persistent restriction of trunk mobility or inability to resume activities of daily living beyond the immediate perioperative period.

No cases of clinically significant graft resorption, columellar skin necrosis, or delayed surgical site infection were identified at any follow-up interval. No patient required intraoperative or postoperative conversion to an alternative structural fixation technique.

## 4. Discussion

The present study introduces a novel technical approach to augmentation rhinoplasty that was developed to address two commonly recognized limitations of conventional costal cartilage techniques: donor-site morbidity and iatrogenic restriction of postoperative nasal tip mobility [1]. The pyramidal columellar strut graft with half-harvest technique represents a methodical integration of established anatomical principles and biomechanical reasoning within the operative framework of costal cartilage rhinoplasty.

Costal cartilage remains the preferred graft material when substantial structural support is required, particularly in revision rhinoplasty and in patients with insufficient septal cartilage [2,4]. Previous studies have demonstrated the versatility and long-term durability of costal cartilage in dorsal augmentation, tip support, and complex reconstructive procedures [3,11]. Nevertheless, concerns regarding postoperative tip rigidity and donor-site morbidity continue to represent important limitations of conventional costal cartilage techniques. The present technique was developed in response to these commonly encountered clinical concerns while maintaining the structural advantages associated with autologous rib cartilage grafting.

The floating-tip columellar strut concept—wherein the inferior end of the strut is seated at the anterior nasal spine without rigid suture attachment to the caudal septum—has been advocated as a method of preserving a greater degree of postoperative tip flexibility while maintaining structural support [15]. Compared with previously described septum-anchored constructs, the present technique was designed to permit a greater degree of dynamic tip movement; however, no direct comparative analysis was performed [6,7]—such as the septal extension graft, which effectively abolishes the rotational freedom of the medial crura—the floating-tip configuration permits physiologic tip depression and elastic recoil under dynamic functional loads [16,17]. The pyramidal strut design introduced in this study advances this principle further by incorporating a graft geometry that anatomically mirrors the native anterior taper of the columellar column, thereby serving as a practical surgical design intended to approximate the native columellar taper while providing basal support and a narrower superior segment suitable for placement between the medial crura.

Septal extension grafts remain among the most reliable techniques for controlling tip projection and rotation and continue to represent a cornerstone of modern structural rhinoplasty [6,8]. However, the degree of rigidity associated with long-term fixation may not be desirable in all patients. Accordingly, the present technique was developed as an alternative option for selected patients in whom preservation of a softer and more natural tip feel was considered clinically relevant. Importantly, no direct comparison with septal extension grafts was performed, and therefore no conclusions regarding relative superiority can be drawn from the present study.

An additional consideration in the design of the pyramidal strut was surgical reproducibility. Because the geometry of the graft follows the natural tapering morphology of the columellar complex, the carving process can be standardized and reproduced with relative consistency. Although objective comparisons with alternative graft designs were not performed, the authors found that the pyramidal configuration facilitated stable positioning within the columellar pocket while maintaining a favorable contour profile.

The mortise-and-tenon engagement between the triangular basal notch of the strut and the bony profile of the anterior nasal spine was intended to enhance positional stability at the graft-spine interface. This interlocking configuration was intended to improve positional stability at the graft-spine interface. Although favorable clinical observations were obtained in this series, objective biomechanical validation was not performed Analogous interlocking base geometries have been described in the context of columellar strut fixation to the anterior nasal spine in septum-anchored rhinoplasty frameworks [18]. By applying this geometric principle within a floating, non-septum-fixed construct, the present technique may provide a balance between basal stability and distal tip flexibility that is without requiring rigid septal fixation.

Therefore, the proposed stabilizing effect of this configuration should be interpreted as a surgical rationale rather than a demonstrated biomechanical advantage. Future cadaveric investigations, finite-element modeling, and mechanical testing studies may further clarify the contribution of this design feature to graft stability.

The half-harvest technique may represent a useful alternative approach for reducing donor-site morbidity of this report. Standard full-thickness costal cartilage harvest requires complete transection of the cartilaginous rib, generating two independently mobile segments whose relative micro-motion during routine trunk movement—particularly rotational and flexural loading—a plausible contributor to postoperative donor-site discomfort, although this mechanism was not directly tested in the present study [19,20].

The half-harvest technique constitutes a central innovation of this procedure. In contradistinction to conventional full-thickness rib harvest, only the medial half of the costal cartilage column was resected; the lateral half cortex and the lateral perichondrial layer were intentionally preserved. This unicortical resection strategy confers three distinct biomechanical and biological advantages.

First, retention of the perichondrium on the residual cartilage surface is known to has been reported to possess chondrogenic potential in experimental studies, with the potential to restore donor-site volume during the remodeling phase. Second, preservation of lateral cortical continuity maintains structural continuity of the rib segment during routine daily activities involving trunk rotation, lateral bending, and sit-to-stand transitions. Trunk rotation refers to rotational movements of the torso around the spinal axis, such as turning the upper body from side to side while the pelvis remains relatively stationary, a maneuver commonly used in clinical assessment of postoperative donor-site discomfort and functional recovery. By avoiding complete transection of the costal cartilage, the technique may theoretically reduce stress concentration at the harvest site; however, this proposed mechanism was not directly evaluated in the present study. Third, the absence of complete intercartilaginous discontinuity enables immediate postoperative trunk mobility, permitting patients to resume activities of daily living, including moderate physical exertion, without the prolonged convalescence characteristic of standard full-thickness harvest.

Conventionally, harvesting costal cartilage grafts frequently induces significant donor-site pain, often compromising upper body mobility and severely restricting the patient’s trunk movement during the first postoperative week. In contrast, our refined technique focuses on minimizing surgical trauma to the surrounding intercostal muscles and preserving the structural continuity of the adjacent cartilage framework. Consequently, this approach significantly mitigates donor-site morbidity, allowing patients to tolerate mechanical stress early on. To clinically verify this immediate functional stability, the senior surgeon routinely performed a manual Trunk-Rotation Test prior to discharge, gently twisting the patient’s upper body left and right while holding their hands. The vast majority of patients demonstrated an absence of sharp donor-site pain and successfully returned to normal activities of daily living within 24 h. Notably, even professional fitness trainers within this cohort were able to safely return to their gym routines within one week post-surgery, highlighting the clinical advantage of this technique in preserving immediate trunk mobility.

Interest in minimizing donor-site morbidity has increased substantially as the use of autologous costal cartilage has expanded. Previous investigations have emphasized postoperative pain, contour deformity, and patient concerns regarding chest wall scarring as important considerations during graft selection. Accordingly, techniques that preserve native rib architecture while providing adequate graft volume may offer practical advantages in selected patients. Whether partial-thickness harvest techniques confer measurable clinical benefits over conventional full-thickness harvest methods remains an important topic for future investigation.

Perichondrial preservation may theoretically contribute to cartilage regeneration, although this was not directly evaluated in the present study. Experimental studies have reported chondrogenic potential within preserved perichondrium; however, donor-site regeneration was not directly evaluated in the present study and therefore should be regarded as a theoretical consideration rather than a demonstrated clinical outcome [21,22]. Although the clinical magnitude of this regenerative phenomenon in the specific context of partial costal cartilage harvest has not yet been prospectively quantified in humans, its theoretical contribution to donor-site volumetric restoration over the medium and long term represents a meaningful additional argument in favor of the half-harvest approach.

The biological significance of perichondrial preservation remains an area of ongoing investigation. Experimental studies have demonstrated the presence of chondrogenic progenitor cells within the perichondrium and have suggested a potential role in cartilage repair and remodeling [23]. However, the extent to which these observations translate into clinically meaningful regeneration following costal cartilage harvest remains uncertain. Consequently, any regenerative benefit associated with the present technique should be interpreted cautiously until validated by dedicated clinical studies.

The multilayer soft tissue coverage protocol employed in this series warrants specific discussion. The characteristically thin and relatively inelastic skin of the nasal tip and columella renders any underlying cartilaginous construct susceptible to visible surface irregularities, edge telegraphing, and progressive contour deformity as the overlying soft tissue undergoes atrophy over time. The application of a perichondrial enveloping layer—supplemented by autologous dermal fat when deemed necessary—provides a compliant biological buffer that attenuates graft surface irregularities, facilitates early fibrovascular integration between the construct and its surrounding tissue bed, and may mitigate the long-term risk of skin thinning and graft prominence.

The postoperative immobilization protocol is a critical component of this technique and demands particular emphasis in preoperative patient counseling. The long-term positional stability of the floating-tip columellar strut is contingent upon progressive fibrovascular integration and the development of a stable soft tissue adherence interface—neither of which is instantaneous. Premature mechanical loading of the nasal tip prior to the establishment of adequate graft-tissue adhesion during the early healing phase poses a substantive and specific risk of graft displacement, axial malrotation, and consequent tip asymmetry. Accordingly, structured postoperative protection of the nasal tip was emphasized during the early recovery period and should be clearly discussed with patients as part of preoperative counseling.

From a clinical perspective, the present technique may be particularly applicable in patients requiring substantial structural augmentation while simultaneously expressing concern regarding postoperative tip rigidity or donor-site morbidity. Because the technique utilizes conventional autologous costal cartilage grafting principles and does not require specialized implants or proprietary devices, it can be incorporated into existing rhinoplasty workflows without substantial modification. Furthermore, the technique should be viewed as an additional option within the spectrum of structural rhinoplasty strategies rather than a replacement for established methods such as septal extension grafting.

Several limitations inherent to the present study merit transparent acknowledgment. The retrospective study design and the absence of a prospective concurrent control cohort preclude definitive comparative inferences regarding nasal tip mobility or donor-site outcomes relative to conventional full-thickness harvest or septum-fixed fixation techniques. Objective quantification of nasal tip mobility using validated dynamometric instruments or three-dimensional motion capture analysis was not incorporated, and functional outcome assessment relied upon clinical examination and standardized photographic documentation—methodologies that, while clinically pragmatic, are subject to inter-observer variability. The single-surgeon, single-center design introduces potential performance bias and constrains the external generalizability of the findings. Prospective randomized controlled trials incorporating validated, objective biomechanical outcome instruments are necessary to rigorously substantiate the clinical observations reported in this series.

Additional limitations should be acknowledged. First, the retrospective design and absence of a control group prevent definitive conclusions regarding superiority over conventional septal extension grafts or full-thickness costal cartilage harvest techniques. Second, postoperative tip flexibility was assessed clinically by the operating surgeon without validated objective instruments, dynamic motion analysis, or patient-reported outcome measures such as FACE-Q [24]. Third, the mean follow-up duration of 8 months limits assessment of long-term graft stability, warping, resorption, and maintenance of tip flexibility. Finally, the private-practice nature of a substantial portion of the cohort may have contributed to loss to follow-up and underestimation of the true long-term revision rate. In addition, because the pyramidal strut configuration, floating fixation strategy, and half-harvest donor technique were applied simultaneously, the independent contribution of each component to the observed outcomes could not be determined. Furthermore, the proposed biomechanical advantages of the pyramidal design and the potential regenerative effects of perichondrial preservation were not directly evaluated and therefore remain theoretical.

Future comparative studies incorporating objective motion analysis, validated patient-reported outcome measures, and longer follow-up durations will be necessary to further define the role of this technique within contemporary costal cartilage rhinoplasty.

## 5. Conclusions

The pyramidal-shaped costal cartilage columellar strut graft with the half-harvest technique represents a feasible surgical option for augmentation rhinoplasty. In this retrospective single-surgeon series, the technique was associated with favorable clinical observations regarding tip flexibility, low donor-site morbidity, and a low incidence of major complications. However, the absence of objective mobility measurements, a control group, and long-term standardized follow-up limits definitive conclusions. Prospective studies incorporating validated outcome measures are warranted to further evaluate the reproducibility, long-term stability, and clinical applicability of this approach.

## Figures and Tables

**Table 1:** Summary of Patient Demographics, Operative Data, and Clinical Outcomes.

Parameter	Value
Total patients,	200
Sex (female/male), (%)	192 (96%)/8 (4%)
Mean age, years (range)	34.2 ± 8.4 (18–65)
Surgery type (primary/revision), (%)	87 (43.5%)/113 (56.5%)
Mean follow-up, months (range)	8.2 ± 3.1 (6–48)
Mean strut length, cm (range)	2.5 ± 0.4 (2.0–5.0)
Adjunctive cap graft, (%)	150 (75%)
Adjunctive shield graft, (%)	89 (44.5%)
Combined cap and shield graft, (%)	87 (43.5%)
Dermal fat graft, (%)	30 (15.0%)
Superficial temporal fascial graft, (%)	102 (51.0%)
Satisfactory tip projection maintained, (%) [95% CI]	179 (89.5%) [85.3–93.8%]
Mild tip deviation, no intervention, (%) [95% CI]	4 (2.0%) [0.5–5.0%]
Revision surgery for tip asymmetry, (%) [95% CI]	1 (0.5%) [0.01–2.7%]
Donor-site complications (pneumothorax/fracture),	0
Infection or graft exposure,	0
Return to daily activities ≤1 week, (%)	200 (100%)

## Data Availability

The data presented in this study are available on request from the corresponding author. The data are not publicly available due to privacy restrictions.

## Supplementary Materials

The following supporting information can be downloaded at: https://www.mdpi.com/article/10.3390/jcm15134985/s1, Video S1: Intraoperative demonstration of pyramidal strut positioning and floating-tip configuration. Following preparation of the anterior nasal spine (ANS) and fixation of the basal portion of the pyramidal costal cartilage strut, multidirectional movement of the distal portion of the construct is demonstrated. The video illustrates the intended floating-tip design concept, in which the graft is securely supported at its base while avoiding rigid fixation of the distal tip complex. This configuration was designed to provide structural support and maintain clinically observable tip flexibility. Video S2: Clinical demonstration of tip flexibility at 12 months following augmenta-tion rhinoplasty using the pyramidal costal cartilage columellar strut with the half-harvest technique. Manual examination demonstrates observable tip movement and elas-tic recoil during postoperative follow-up. These clinical observations are consistent with the intended design characteristics of the floating-tip fixation strategy and suggest maintenance of tip flexibility during the follow-up period. However, objective biomechan-ical assessment and quantitative motion analysis were not performed.
