# Supplementary material for: Pyramidal-Shaped Costal Cartilage Columellar Strut Graft with Half-Harvest Technique for Augmentation Rhinoplasty: A Novel Approach to Tip Mobility Preservation
Source: J Clin Med. 2026 Jun 26;15(13):4985. doi: 10.3390/jcm15134985 (PMC13362741; doi:10.3390/jcm15134985)
Supplement: Supplementary file 1 [file jcm-15-04985-s001.zip › jcm-4361071-supplementary.pdf]

SUPPLEMENTARY DIGITAL CONTENT

Pyramidal-Shaped Costal Cartilage Columellar Strut Graft With Half-Harvest Technique for Augmentation Rhinoplasty: A Novel Approach to Tip Mobility Preservation

Hyo Heon Kim, MD, PhD; Hee Jun Son, MD

Supplemental Digital Content S1 — VIDEO S1

Title: Intraoperative Demonstration of Pyramidal Columellar Strut Mobility

Intraoperative demonstration of pyramidal strut positioning and floating-tip configuration. Following preparation of the anterior nasal spine (ANS) and fixation of the basal portion of the pyramidal costal cartilage strut, multidirectional movement of the distal portion of the construct is demonstrated. The video illustrates the intended floating-tip design concept, in which the graft is securely supported at its base while avoiding rigid fixation of the distal tip complex. This configuration was designed to provide structural support and maintain clinically observable tip flexibility.

Note to authors: Please upload Video S1 as a separate video file (MP4 or MOV preferred, maximum 5 minutes). This legend will be linked to Supplemental Digital Content 1 upon acceptance and publication online

at www.mdpi.com/journal/jcm.

Supplemental Digital Content S2 — VIDEO S2

Title: Postoperative Nasal Tip Mobility at 12 Months Following Surgery

Clinical demonstration of tip flexibility at 12 months following augmenta-tion rhinoplasty using the pyramidal costal cartilage columellar strut with the half-harvest technique. Manual examination demonstrates observable tip movement and elas-tic recoil during postoperative follow-up. These clinical observations are consistent with the intended design characteristics of the floating-tip fixation strategy and suggest maintenance of tip flexibility during the follow-up period. However, objective biomechan-ical assessment and quantitative motion analysis were not performed.

Note to authors: Please upload Video S2 as a separate video file (MP4 or MOV preferred, maximum 5 minutes). This legend will be linked to Supplemental Digital Content 2 upon acceptance and publication online at www.mdpi.com/journal/jcm.
